# Draft genome sequence of *Paraclostridium benzoelyticum* strain YGCA10, isolated from sediment of the Etoliko lagoon in Greece

**DOI:** 10.1128/mra.00477-24

**Published:** 2024-08-27

**Authors:** Ioannis Galiatsatos, Elias Asimakis, Panagiota Stathopoulou, George Tsiamis

**Affiliations:** 1 Department of Sustainable Agriculture, University of Patras, Agrinio, Greece; California State University San Marcos, San Marcos, California, USA

**Keywords:** lagoon sediment, *Paraclostridium benzoelyticum*, anaerobes, bioprospecting

## Abstract

*Paraclostridium benzoelyticum* is a Gram-positive, heterotrophic, and obligately anaerobic bacterial species. This report describes the draft genome sequence of *P. benzoelyticum* strain YGCA10, isolated from lagoon sediment under anaerobic culture conditions.

## ANNOUNCEMENT

The Etoliko lagoon is an extreme environment that is characterized by anoxic conditions in the hypolimnion and high sulfate concentrations (1). Despite these conditions, the lagoon supports a diverse range of microorganisms, including putative new bacterial and archaeal phyla (2). Bioprospecting this ecosystem may yield interesting extremophiles, with high potential for exploitation in biotechnological applications. In this context, the genome of a Gram-positive, strictly anaerobic *Paraclostridium benzoelyticum* strain (3) isolated from the deep sediment of the lagoon was sequenced.

A sediment core (70-cm height, 5 cm wide) was extracted from the lagoon bed (depth 26.8 m) and stored at −80°C. Homogenates were prepared by mixing 2 g of frozen sediment (extracted from the middle of the core) with 12 mL of 1× phosphate-buffered saline solution. All procedures were carried out under anaerobic conditions using a mixture of 70% vol/vol N_2_ and 30% vol/vol CO_2_. Then, 2.5 mL of the homogenate was inoculated in 12 mL of a liquid medium enriched in salt and minerals (4) in 30-mL glass vials. The vials were sealed and incubated at 25°C for 2 weeks. A 100-µL inoculum was spread on Luria-Bertani (LB) agar plates and incubated in BD Gaspack EZ container systems (Becton Dickinson, New Jersey, USA) at 25°C for 2 weeks. Single colonies were restreaked three times onto LB agar and incubated under the same conditions.

Genomic DNA extraction was performed on a single colony (third restreak) using a modified CTAB (cetyl-trimethylammonium bromide) protocol (5) with 1-hour lysis time. Libraries were prepared with the Ligation Sequencing Kit v.14 according to the manufacturer’s instructions (Oxford Nanopore Technologies, Oxford, UK) using 1 µg of genomic DNA. The long fragment buffer was used to enrich the library with DNA fragments of 3 kb or longer. Sequencing was performed on a minION platform (R10.4.1 flow cell) for 72 hours. Dorado v.0.6.0 (6) was used for basecalling and NanoFilt (v.2.8.0–1) (7) for filtering the reads based on a quality control (QC) threshold set above 10. *De novo* read assembly was carried out with Flye v.2.9.3 (8), and the quality of assemblies was assessed with QUAST v.5.2.0 (9). Genome annotation was performed with National Center for Biotechnology Information’s Prokaryotic Genome Annotation Pipeline (10). Genome completeness and contamination were assessed with CheckM v.1.2.2 (11). Taxonomic classification was assigned with the Genomes Taxonomy Database (GTDB)-Toolkit v.2.3.2 (12) based on GTDB (13). Average nucleotide identity (ANI) was determined with FastANI v.1.34 (14). Default parameters were used except where otherwise noted.

Almost 2.6 Gb of 601-k reads with N_50_ equal to 11.28 kb was produced after sequencing. The mean read quality was estimated at QC 11.1. The assembled draft genome consisted of five contigs with a total length of 3.6 Mb, resulting in 630× sequencing coverage and a GC content of 28.5% as calculated with QUAST v.4.4 (15). The largest contig was 3.27 Mb in length, which was also the *N*_50_ of the assembly. Similarly, *L*_50_ was calculated at one contig. Genome completeness was estimated at 96.14%. Genome annotation revealed the presence of 4,131 genes. These included 3,586 protein-coding genes, 873 of which were coding for hypothetical proteins, 387 pseudogenes, and 158 RNA genes. The genomic sequence was classified as *P. benzoelyticum*, showing 99.07% ANI (ASM100628v1) with said species.

## Data Availability

The Whole Genome Shotgun project for *P. benzoelyticum* strain YGCA10 has been deposited at DDBJ/ENA/GenBank under accession no. JBCLWQ000000000. The genomic sequences are part of a BioProject with accession number PRJNA1105726, and a BioSample with accession number SAMN41108139. Raw sequencing reads were deposited in the Sequence Read Archive under accession number SRP504839. The version described in this paper is version JBCLWQ010000000.
